# Coexisting nephrotic syndromes influences in st elevation myocardial infarction patient and chronic limb-threatening ischemia patient: is there any correlation?

**DOI:** 10.12688/f1000research.134021.1

**Published:** 2023-05-23

**Authors:** Iwan Dakota, Taofan Taofan, Suci Indriani, Jonathan Edbert Afandy, Mikhael Asaf, Swastya Dwi Putra, Suko Adiarto, Renan Sukmawan

**Affiliations:** 1 Department of Cardiology and Vascular Medicine, Faculty of Medicine University of Indonesia / National Cardiovascular Center Harapan Kita / University of Indonesia Academic Hospital, Jakarta, Indonesia; 2 Assistant of Vascular Division, Department of Cardiology and Vascular Medicine, Faculty of Medicine University of Indonesia / National Cardiovascular Center Harapan Kita / University of Indonesia Academic Hospital, Jakarta, Indonesia; 3 Cardiology Resident, Department of Cardiology and Vascular Medicine, Faculty of Medicine University of Indonesia / National Cardiovascular Center Harapan Kita / University of Indonesia Academic Hospital, Jakarta, Indonesia

**Keywords:** nephrotic syndrome, acute coronary syndrome, STEMI, peripheral artery disease, chronic limb-threatening ischemia, thromboembolism, young adult

## Abstract

**Background:** ST elevation myocardial infarction (STEMI) and chronic limb-threatening ischemia (CLTI) were cardiovascular emergencies and require urgent reperfusion treatment. Both diseases shared same traditional cardiovascular risk factors. Nephrotic syndrome (NS) patients were known for risk of thromboembolic complications that may present as STEMI or CLTI, result of hypercoagulable state stemming leading to thrombus formation.

**Case illustration:** Three cases were described in a case series. The first case presented with anterior extensive STEMI, coroangiography revealed total occlusion at proximal left anterior descending artery with high burden thrombus, treated with defered stenting and medical therapy. The second case presented with CLTI, imaging modality showed occlusion with thrombus in infra-renal abdominal aorta until bilateral superficial femoral artery (SFA), the patient refused any interventional treatment, so he was treated with medical therapy only. The third case presented with CLTI on left leg and chronic limb ischemia on right leg, imaging modality showed occlusion at left external iliac artery and 1/3 distal of right SFA with prominent plaque calcification, treated with percutaneous transluminal angioplasty, and medical therapy. All patients achieved significant improvement in the disease.

**Conclusion:** NS is a risk factor for STEMI and CLTI. Even corticosteroids for NS treatment also a risk factor for thromboembolic complications. Controlling the disease severity with precaution of the therapy side effect should be achieved. If thromboembolic complications related to NS happen, the management mainly follows the available guidelines.

## Introduction

Acute coronary syndromes (ACS) are clinical entity characterized by a sudden reduction in blood supply to the heart and often reflects a degree of damage to the coronary arteries by atherosclerosis; plaque rupture, thrombosis, and inflammation. ^1^^,^^2^ The current classification of ACS depends on Electrocardiogram (ECG) findings at admission: non-ST Elevation Myocardial Infarction and ST Elevation Myocardial Infarction (STEMI) representing the most dangerous form of the pathology and therefore requiring urgent reperfusion treatment. ^3^ Chronic limb-threatening ischemia (CLTI) is a manifestation of peripheral arterial disease that is characterized by chronic, inadequate tissue perfusion at rest. ^4^ CLTI is defined by the presence of peripheral artery disease (PAD) in combination with rest pain or tissue loss (gangrene, ulceration) for more than two weeks duration. ^5^ Both ACS and PAD shared the same traditional cardiovascular risks factors such as advanced age, male sex, smoking, hypertension, diabetes, and dyslipidemia. ^1^^,^^6^

Nephrotic syndrome (NS) is a condition characterized by the presence of peripheral edema, heavy proteinuria, and hypoalbuminemia, often with hyperlipidemia. The syndrome can be due to intrinsic renal disease or secondary to an underlying medical condition. ^7^ Patients with NS have long been assumed to be at increased risk for atherosclerosis and cardiovascular disease because of NS-associated hyperlipidemia and hypertension. ^8^ NS patients were also at risk of thromboembolism that may form in either arteries or veins result of the hypercoagulable state stemming from imbalances in the coagulation cascade leading to thrombus formation that obstructs blood flow. ^8^^,^^9^

Although NS-caused venous thromboembolism is well recognized, arterial thrombosis has rarely been reported. ^10^ This case series aims to describe a case of STEMI and two cases of CLTI in young adults with nephrotic syndrome and how to overcome the disease in National Cardiovascular Center, Harapan Kita, Jakarta, Indonesia.

## Case illustration

### Case 1

A 29-year-old Javanese male presented with chest pain radiating to his left arm followed by sweating, nausea, and vomiting for the last 18 hours. He has been diagnosed with nephrotic syndrome in the past 12 years without any other risk factors such as hypertension, dyslipidemia, diabetes mellitus, smoking, or family history. He consumed steroids for NS but stopped this medicine for the last 2 months.

**Figure 1.**
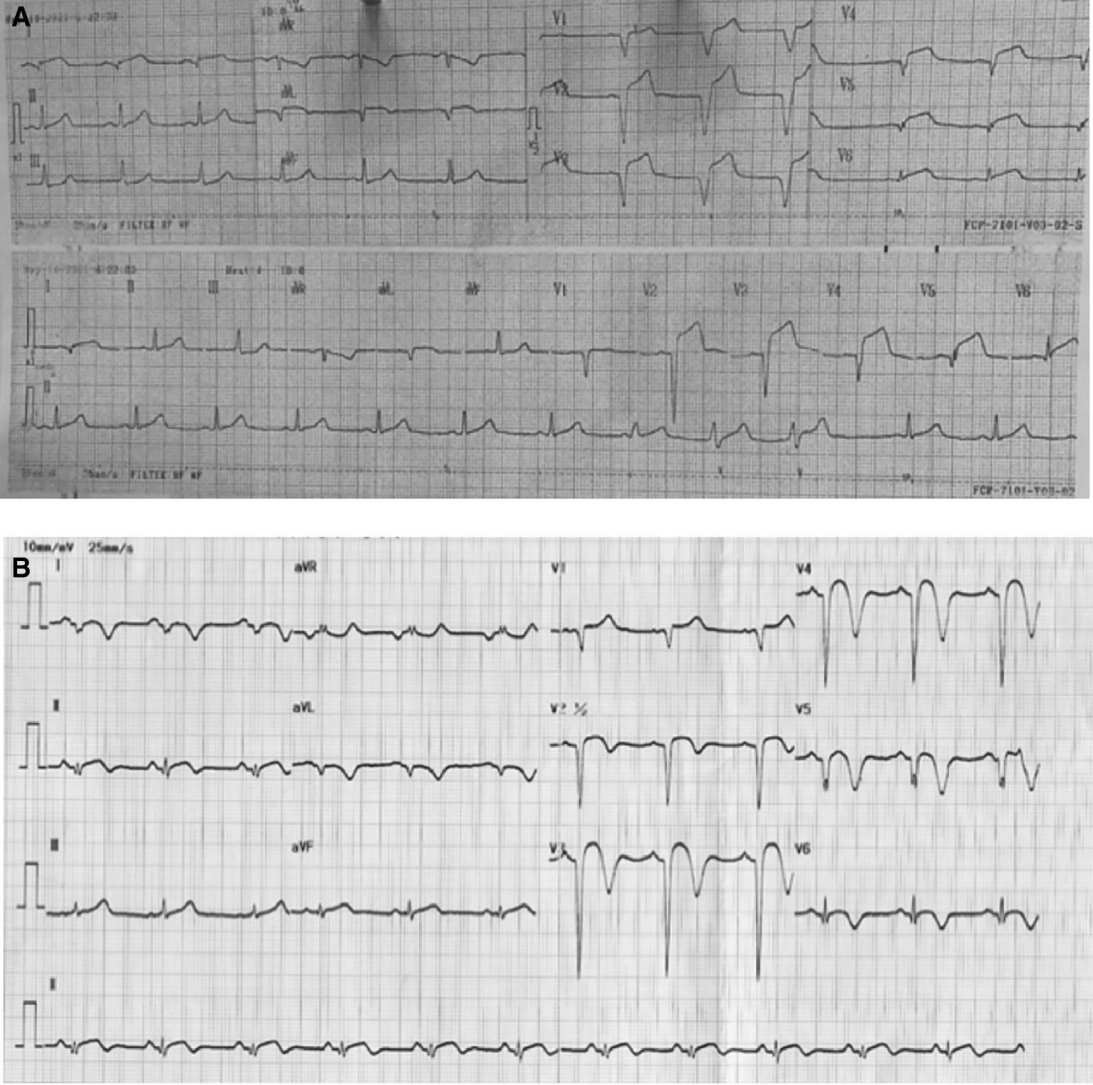
ECG of 1 ^st^ patient. A. After 18 hours onset of chest pain, ST elevation and pathological Q waves were seen in V1-V6, I, and aVL. B. After percutaneous coronary intervention and medical therapy, no dynamic ST-T changes was seen in the ECG.

Physical examination revealed high blood pressure with normal heart rate and fever with temperature of 38°C. Chest auscultation showed crackles in both lungs without any rales or wheezing. ECG on 18 hours of chest pain onset showed ST elevation and pathological Q waves in V1-V6, I, and aVL ( Figure 1A). Laboratory examination showed leukocytosis, high level of high-sensitive troponin T, hypoalbuminemia, proteinuria, and hyperlipidemia. Chest X-ray revealed infiltrate in both lungs. Echocardiography showed reduced left ventricular ejection fraction (LVEF) of 43%, hypokinetic at anterior and lateral segments, and left ventricle thrombus.

**Figure 2.**
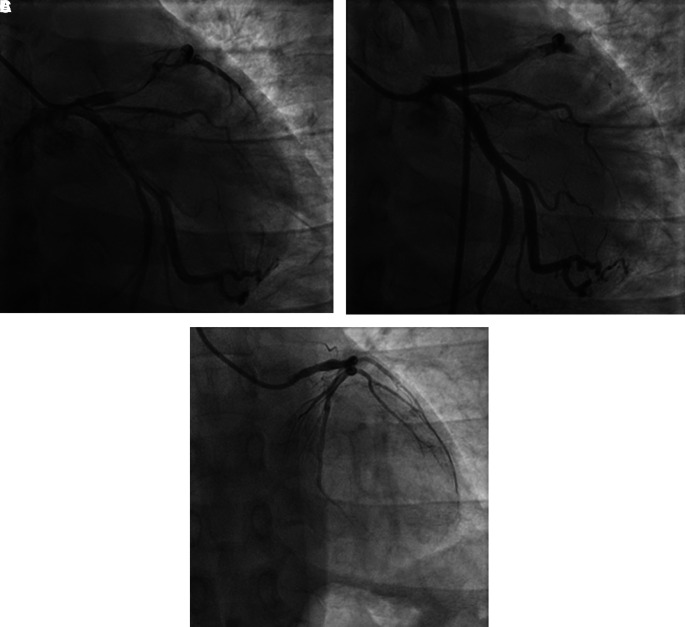
Coroangiography of 1 ^st^ patient. A. Before percutaneous coronary intervention, total occlusion at proximal left anterior descending artery, thrombus grade 5, and TIMI flow 1. B. After percutaneous coronary intervention, TIMI flow 1 with residual thrombus in LAD and shifting thrombus to distal left circumflex artery. C. After 4 months follow-up, normal coronary arteries without any apparent atherosclerotic lesion.

The patient was diagnosed with STEMI anterior extensive, Killip I, TIMI 3/14, nephrotic syndrome, and community-acquired pneumonia. Coroangiography (CAG) revealed total occlusion at proximal left anterior descending (LAD) artery, thrombus grade 5, and TIMI flow 1 ( Figure 2A). The patient was then planned to receive plain old balloon angioplasty (POBA) in LAD. After multiple attempts of extensive POBA, CAG showed TIMI flow 1 with residual thrombus in LAD and shifting thrombus to distal left circumflex artery (LCx) ( Figure 2B). It was decided to defer further maneuvers and proceeded to medical treatment with intravenous antiplatelet infusion and anticoagulan.

The patient received eptifibatide infusion, heparinization, oral dual antiplatelet with aspirin and ticagrelor, ACE inhibitor, statin, nitrate, and antibiotic. On the following day, there was no chest pain and ECG did not show any dynamic ST-T changes ( Figure 1B). The patient was then received steroid therapy and discharged with stable condition. Four months later, without any signs and symptoms, he underwent CAG that showed normal coronary arteries without any apparent atherosclerotic lesions ( Figure 2C).

### Case 2

A 30-year-old Sundanese male presented with a chief complaint of wound in his leg since 6 months ago accompanied by resting pain. At first, he complained of pain in both legs when walking for distance in the past 6 years. The patient had history of NS since 12 years ago, but he didn’t take any medication routinely. He was also a smoker, smoking 1 pack of cigarettes per day. He denied history of hypertension and diabetes mellitus.

**Figure 3.**
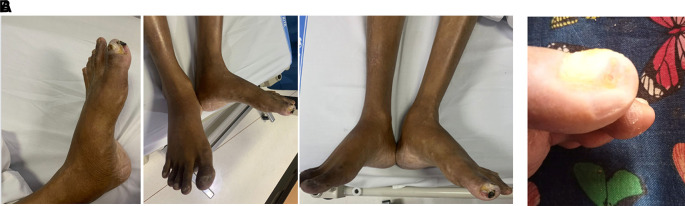
Clinical image of 2 ^nd^ patient. A. Gangrene was seen on the left toe at presentation. B. Resolution of gangrene after 3 weeks follow-up.

**Figure 4.**
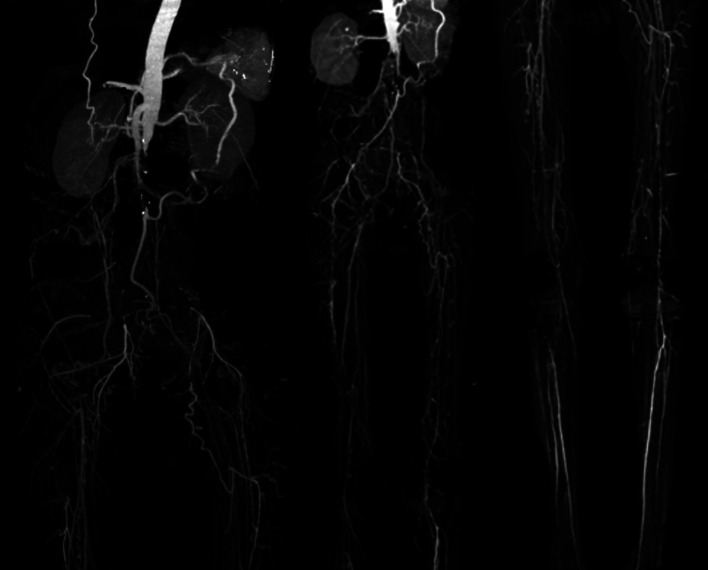
Lower extremity CT Angiography of 2 ^nd^ patient. Occlusion with thrombus in abdominal aorta starting from 2 cm below renal artery until bilateral superficial femoral artery, distal flow filled from collateral from branch of coeliac trunk and branch of superior mesenteric artery.

His vital signs were within normal limits. Physical examination showed cold extremities, non-palpable bilateral dorsalis pedis artery pulsation, and gangrene on left toe ( Figure 3A). Significant laboratory examination results were erythrocyte sedimentation rate of 99 mm/hour, D-dimer of 3250 ng/mL, fibrinogen of 734 mg/dL, albumin of 0.8 g/dL, total cholesterol of 347 g/dL, LDL of 257 g/dL, HDL of 54 g/dL, triglyceride of 278 g/dL, +3 urinary protein with 24-hour urinary protein of 19840 mg/24 hour. Left ankle brachial index (ABI) of the patient was 0.25 and right was 0.33. Lower extremity duplex ultrasound (DUS) was consistent with lower extremity CT-Scan Angiography (CTA) revealed occlusion with thrombus in abdominal aorta starting from 2 cm below renal artery until bilateral superficial femoral artery (SFA), distal flow filled from collateral from branch of coeliac trunk and branch of superior mesenteric artery ( Figure 4).

The patient was diagnosed with CLTI (Rutherford III-5, WIFi Score 2-3-0) in aortio-iliaca occlusive disease, TASC II type D lession, and nephrotic syndrome. The patient unfortunately refused any intervention therapy. Then, he was planned for albumin transfusion, methylprednisolone therapy with titration method, heparinization, clopidogrel, lumbrokinase, simvastatin, diltiazem, candesartan, and some supportive symptomatic medication. After 5 days, the patient was discharged with lesser degree of leg pain. His albumin increased to 2.9 g/dL and better 24-hour urinary protein of 5685 mg/24 hour. His take-home medications were 3 × 16 mg methylprednisolone, 1 × 75 mg clopidogrel 1 × 75mg, 1 × 40 mg simvastatin, 1 × 16 mg candesartan, and 1 × 100 mg diltiazem. Follow-up after 3 weeks showed significant leg pain improvement and resolution of gangrene ( Figure 3B).

### Case 3

A 32-year-old Javanese male presented with chief complaints of leg pain. The pain has been experienced for one year, at first, felt only when walking for distances, but it got worse, and he started to feel resting pain in the past one month. The patient had nine years history of NS confirmed by kidney biopsy with result of focal segmental glomerulosclerosis. He denied history of hypertension, diabetes, or smoking. At presentation, he took 2 × 360 mg mycophenolic acid and 1 × 8 mg methylprednisolone daily.

**Figure 5.**
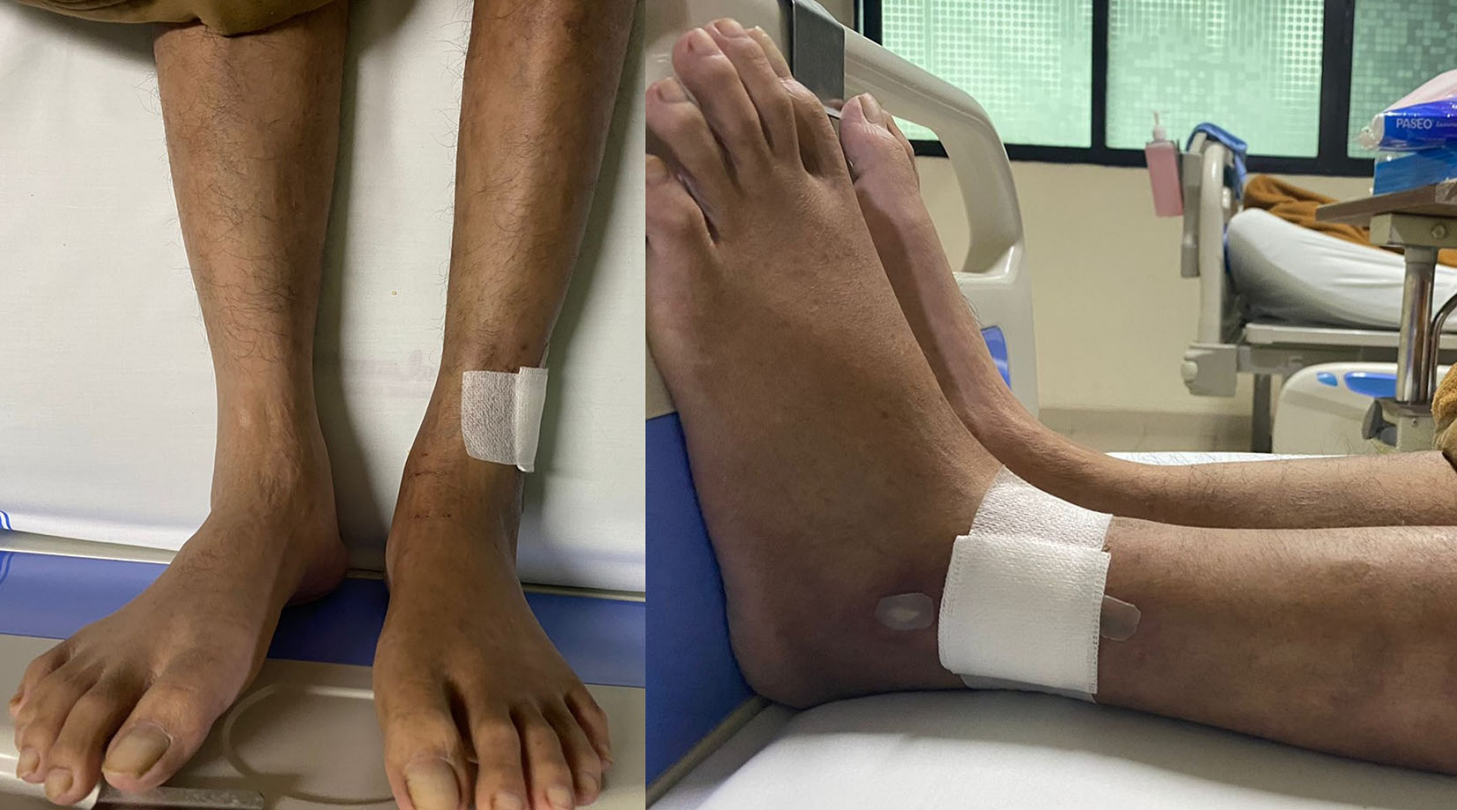
Clinical image of 3 ^rd^ patient. Ulcer (covered by bandage), hair loss, and atrophy were seen on the left leg.

**Figure 6.**
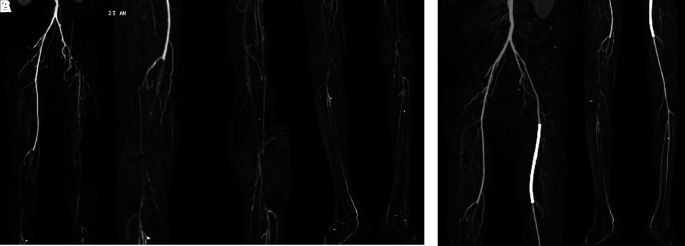
Lower extremity CT Scan Angiography of 3 ^rd^ patient. A. Pre-intervention, occlusion at level of left external iliac artery and 1/3 distal of right superficial femoral artery with prominent plaque calcification. B. Before 2 ^nd^ intervention, positive flow until distal of the left leg with patent stent.

Vital signs were within normal limits. Physical examination revealed ulcer, hair loss, and atrophy on the left leg ( Figure 5). Significant laboratory examination results were D-Dimer of 2990 ng/mL, total cholesterol of 233 g/dL, LDL of 187 g/dL, triglycerides of 164 g/dL, and urine albumin of 413 mg/L. His serum albumin was normal (184 g/dL). His right ABI was 0.5 on left was 0.33. Lower extremity DUS and CTA showed occlusion at level of left external iliac artery and 1/3 distal of right SFA with prominent plaque calcification ( Figure 6A).

**Figure 7.**
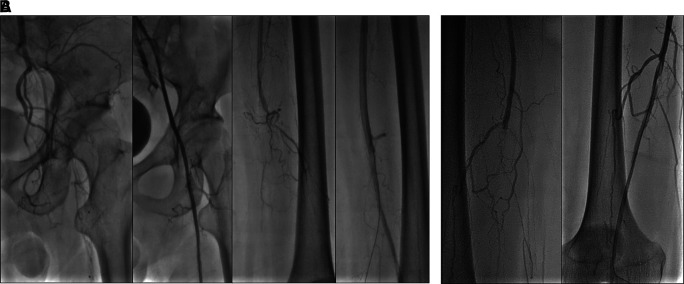
Percutaneous transluminal angioplasty procedure of 3 ^rd^ patient. A. First intervention, contrast flow until distal of left leg artery after percutaneous transluminal angioplasty. B. Second intervention, contrast flow until distal of right leg artery after percutaneous transluminal angioplasty.

The patient’s diagnosis at the time was CLTI with ulcer on the left leg (Rutherford III-5, WIFi Score 1-3-0), chronic limb ischemia on the right leg (Rutherford I-3, WIFi Score 0-2-0), TASC II type D lession, and nephrotic syndrome. He was treated with heparinization and two episodes of percutaneous transluminal angioplasty (PTA). First, with POBA done at left Iliac Artery and SFA with addition of 6.0 × 120 mm drug-eluting stent (DES) overlapped with 6.0 × 80 mm (Boston Scientific, Marlborough, MA, USA) at SFA ( Figure 7A). Second, POBA at mid–distal right SFA 5 months later ( Figure 7B). CTA after the first procedure ( Figure 6B) and angiography after second procedure with lower extremity DUS confirmed positive flow until distal vessel of both lower limbs. The patient was discharged without any complaint and received rivaroxaban, clopidogrel, aspirin, simvastatin, mycophenolic acid, and methylprednisolone as his routine medication. he was also educated to do exercise therapy.

## Discussion

According to a publication by Mahmoodi, et al., ^11^ the annual incidence of arterial thromboembolism rate in NS patients was 1.48%. The most common first ATE presentation in NS patients was myocardial infarction (44%), followed by unstable angina pectoris (14%), peripheral artery disease (14%), ischemic stroke (11.5%), cerebral transient ischemic attack (11.5%), amaurosis fugax (2%), and aorta thrombosis (2%).

The pathophysiology of ATE in NS patients hasn’t known clearly. It was postulated that protein plasma alteration involves coagulation and fibrinolysis disturbance, increased aggregation of platelet, low albumin plasma, hyperviscosity, and dyslipidemia. ^11^^,^^12^ Chronic excessive proteinuria with long-term abnormal hemostasis and lipid profiles such as in NS patients. ^13^ There are three proposed mechanisms related to the hypercoagulable state in NS patients. First, enhanced coagulation related to low molecular weight protein loss such as factors IX, XI, and XII from urine, thereby the liver increased synthesis of factors II, VII, VIII, X, XIII, and fibrinogen to compensate the hypoalbuminemia state. Second, decreased anticoagulation such as Antithrombin III that has been observed in low serum albumin condition. Third, fibrinolytic system imbalance related to decreased levels of plasminogen and raised levels of plasminogen activator that correlate with the degree of hypoalbuminaemia.

Our 1 ^st^ and 2 ^nd^ patient were in acute phase of nephrotic syndrome which may increase the predisposition to develop thromboembolic events with known low levels of serum albumin. Thromboses are frequent at plasma albumin levels <2 g/dl. ^14^ However, our 3 ^rd^ patient had good control of the disease, known from relatively normal serum albumin. We suspected that long-term corticosteroid use by our patient promotes a hypercoagulable state since it increased factors II, V, VII, IX, X, and XII and fibrinogen, thereby increasing risk for thrombosis. ^15^ Hyperlipidemia in all of our patients is also known to be a risk factor for thrombosis since it induced platelet hyperaggregability. ^16^

Currently, there is no consensus according to the management of thromboembolic complications related to NS. ^17^ The management mainly follows the available guidelines and depends on the location and hypercoagulable state. We found that the 1 ^st^, patient possible pathogenesis of myocardial infarction is due to coronary thrombosis which is shown by high burden thrombus (HTB) in CAG. Publication by Xie, et al. ^13^ supported our findings by showing that most NS patients CAG identified acute coronary thrombosis rather than atheromatous plaque. A thrombus in coronary artery with a score of ≥4 is defined as high thrombus burden (HTB), which deferred stent placement has been associated with a better outcome. ^18^ Pharmacological therapies that are used for HTB treatment include antiplatelet, anticoagulant, thrombolytic, statins, and vasodilators.

In CLTI, existing evidence argues strongly for selective revascularization based on specific clinical and anatomical criteria for optimal treatment. ^5^ Endovascular intervention in CLTI relies upon the ability to cross the Femoro-Popliteal lesion, including techniques for vessel preparation and definitive therapy. ^19^ Unfortunately, there is only a small number of publications for guidance to choose specific endovascular techniques for CLTI patients. CLTI patients are recommended to receive pharmacological therapy with antiplatelet and moderate-to-high-intensity statin therapy to reduce the risk of major adverse cardiovascular events. For patients that are not suitable for revascularization, there are few options for non revascularization interventions, pharmacotherapy, and conservative management. We would like to choose endovascular approach for our 2 ^nd^ and 3 ^rd^ patient, however, the 2 ^nd^ patient refused any intervention, so we optimized the pharmacological therapy. Both of our patients achieved significant improvement in the disease.

Treatment of NS patients with immunosuppressive therapy combined with steroids can reduce disease activity, which reduced approximately 40% risk of progression to end-stage renal disease compared to no treatment or supportive treatment alone. ^20^ Prophylaxis for thromboembolism also can be given to NS patients depending on histological subtype, bleeding risk, and serum albumin level, which are received by our patients. ^21^

## Conclusion

We’ve reported three cases of NS-caused arterial thromboembolism complication in young patient. NS is a risk factor for STEMI and CLTI due to thrombosis and/or atherosclerotic processes. Even corticosteroids for NS treatment also induce a hypercoagulable state and become risk factor for thromboembolic complications. Controlling the disease severity with precaution of the therapy side effect should be achieved. If thromboembolic complications related to NS happen, the management mainly follows the available guidelines.

## Consent

Written informed consent for publication of their clinical details and clinical images was obtained from the patients.
