# Coexisting nephrotic syndromes influences in st elevation myocardial infarction patient and chronic limb-threatening ischemia patient: is there any correlation?

**DOI:** 10.12688/f1000research.134021.2

**Published:** 2025-03-31

**Authors:** Iwan Dakota, Taofan Taofan, Suci Indriani, Jonathan Edbert Afandy, Mikhael Asaf, Swastya Dwi Putra, Suko Adiarto, Renan Sukmawan

**Affiliations:** 1 Department of Cardiology and Vascular Medicine, Faculty of Medicine University of Indonesia / National Cardiovascular Center Harapan Kita / University of Indonesia Academic Hospital, Jakarta, Indonesia; 2 Assistant of Vascular Division, Department of Cardiology and Vascular Medicine, Faculty of Medicine University of Indonesia / National Cardiovascular Center Harapan Kita / University of Indonesia Academic Hospital, Jakarta, Indonesia; 3 Cardiology Resident, Department of Cardiology and Vascular Medicine, Faculty of Medicine University of Indonesia / National Cardiovascular Center Harapan Kita / University of Indonesia Academic Hospital, Jakarta, Indonesia

**Keywords:** nephrotic syndrome, acute coronary syndrome, STEMI, peripheral artery disease, chronic limb-threatening ischemia, thromboembolism, young adult

## Abstract

**Background:**

ST-elevation myocardial infarction (STEMI) and chronic limb-threatening ischemia (CLTI) are severe cardiovascular emergencies requiring urgent intervention. Nephrotic syndrome (NS) increases the risk of arterial thromboembolism (ATE), but its exact contribution remains underrecognized.

**Case illustration:**

We present three cases of young adults with NS who developed ATE. The first patient had anterior STEMI with high thrombus burden but no significant atherosclerosis, suggesting a thromboembolic event. The second patient, diagnosed with CLTI, had extensive thrombotic occlusions from the infrarenal aorta to the bilateral superficial femoral arteries without atherosclerotic plaques, reinforcing a thromboembolic mechanism. He declined revascularization and was treated with medical therapy, achieving symptom relief. The third patient had CLTI with occlusions in the external iliac and superficial femoral arteries, accompanied by prominent plaque calcification, suggesting an atherosclerotic contribution. He underwent percutaneous transluminal angioplasty with favorable outcomes.

**Conclusion:**

NS predisposes patients to ATE via hypercoagulability and, in some cases, atherosclerosis. Cardiovascular screening should be prioritized in high-risk patients, and preventive measures, including thromboprophylaxis and lipid management, should be considered. Treatment should be individualized based on the predominant mechanism, with deferred stenting in high thrombus burden STEMI and a multidisciplinary approach for CLTI. Long-term follow-up is essential to prevent recurrence.

## Introduction

Acute coronary syndromes (ACS) encompass a group of clinical conditions characterized by a sudden reduction in blood supply to the heart and often due to underlying atherosclerosis, plaque rupture, thrombosis, and inflammation. ^1^^,^^2^ The current classification of ACS is based on electrocardiogram (ECG) findings at admission with ST Elevation Myocardial Infarction (STEMI) representing the most severe form of requiring urgent reperfusion treatment. ^3^ Chronic limb-threatening ischemia (CLTI) is a manifestation of peripheral arterial disease (PAD) characterized by chronic, inadequate tissue perfusion at rest. ^4^ It defined by the presence of peripheral artery disease alongisde rest pain or tissue loss (gangrene, ulceration) lasting more than two weeks duration. ^5^ Both ACS and PAD share the same traditional cardiovascular risks factors such as advanced age, male sex, smoking, hypertension, diabetes, and dyslipidemia. ^1^^,^^6^

Nephrotic syndrome (NS) is a condition marked by the presence of peripheral edema, heavy proteinuria, and hypoalbuminemia, often with hyperlipidemia. The syndrome can be due to intrinsic renal disease or secondary to an underlying medical condition. ^7^ Patients with NS are assumed to be at increased risk for atherosclerosis and cardiovascular disease due to NS-associated hyperlipidemia and hypertension. ^8^ Additionally, they are predisposed to thromboembolism due to a hypercoagulable state caused by imbalances in the coagulation cascade, leading to thrombus formation and vascular obstruction. ^8^^,^^9^

While venous thromboembolism in NS is well recognized, arterial thromboembolism (ATE) is rarely been reported. ^10^ This case series aims to describe a case of STEMI and two cases of CLTI in young adults with NS, as well as their management at the National Cardiovascular Center, Harapan Kita, Jakarta, Indonesia.

## Case illustration

### Case 1

A 29-year-old Javanese male presented with chest pain radiating to his left arm accompanied by sweating, nausea, and vomiting for the past 18 hours. He had been diagnosed with NS 12 years ago without any other risk factors such as hypertension, dyslipidemia, diabetes mellitus, smoking, or family history. He had been taking steroids for NS but discontinued them two months ago.

**Figure 1.**
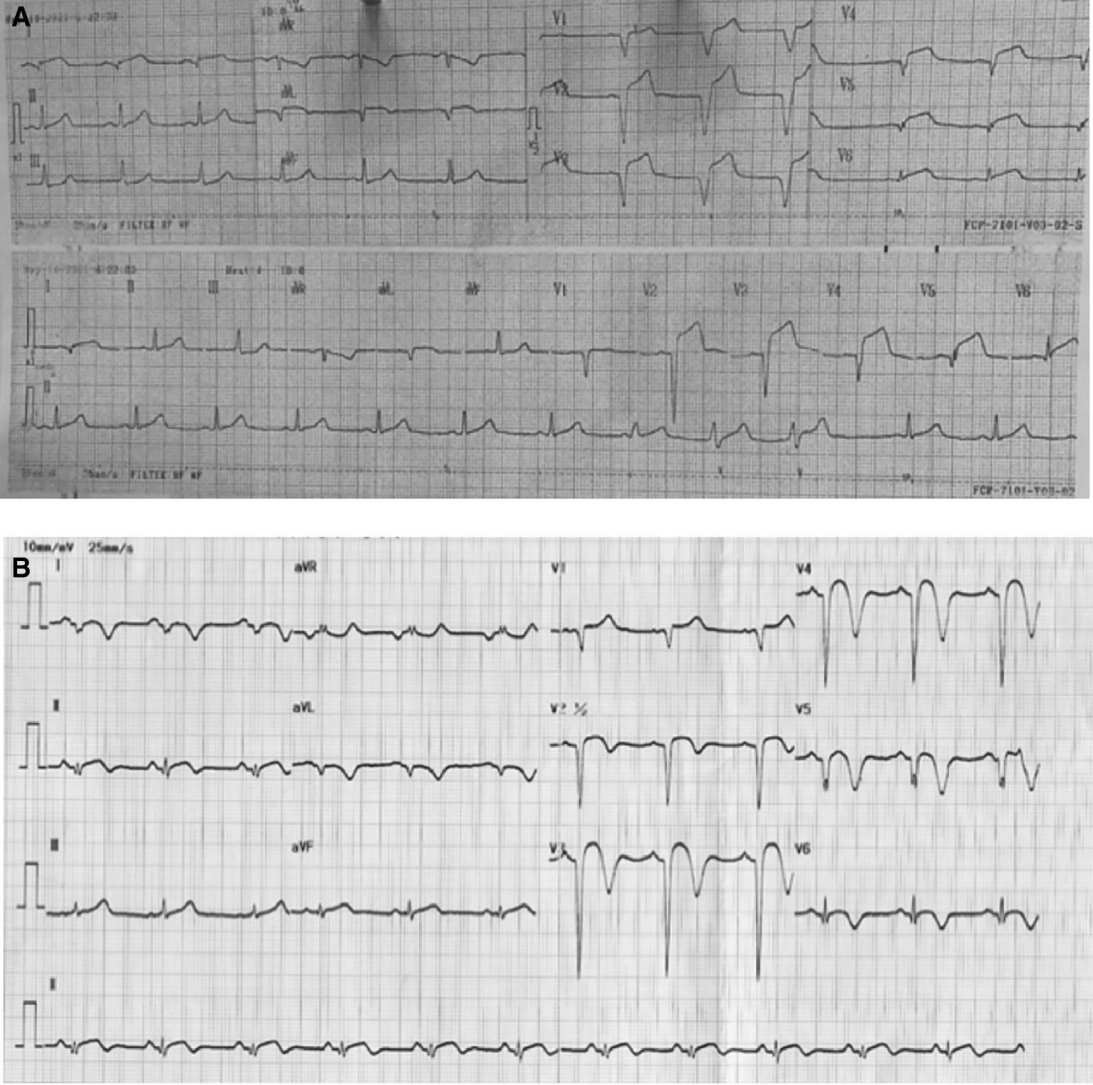
ECG of 1 ^st^ patient. A. After 18 hours onset of chest pain, ST elevation and pathological Q waves were seen in leads V1-V6, I, and aVL. B. After percutaneous coronary intervention and medical therapy, no dynamic ST-T changes was seen in the ECG.

On physical examination, he had elevated blood pressure, a normal heart rate, and fever with temperature of 38°C. Chest auscultation revealed crackles in both lungs without rales or wheezing. An ECG performed 18 hours after symptom onset showed ST elevation and pathological Q waves in leads V1-V6, I, and aVL ( Figure 1A). Laboratory findings included leukocytosis, elevated high-sensitive troponin T, hypoalbuminemia, proteinuria, and hyperlipidemia. A Chest X-ray showed bilateral pulmonary infiltrates. Echocardiography revealed a reduced left ventricular ejection fraction (LVEF) of 43%, hypokinetic in the anterior and lateral segments, and the presence of left ventricular thrombus.

**Figure 2.**
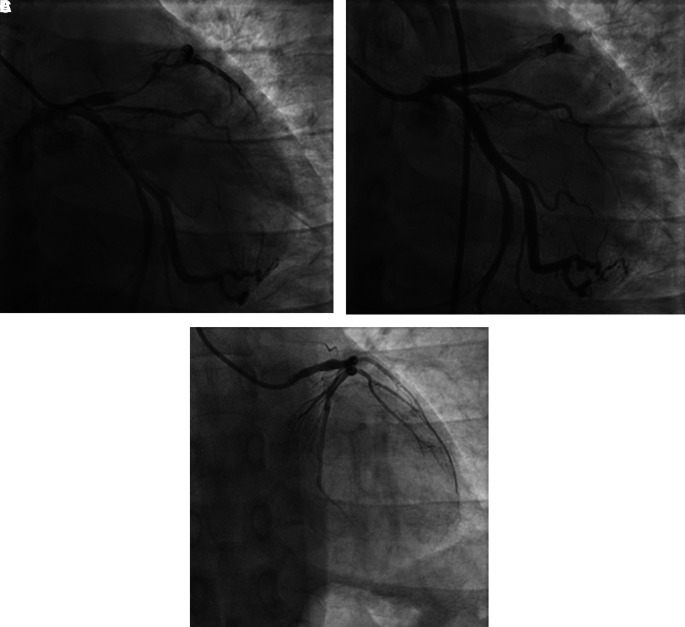
Coronary angiography of 1 ^st^ patient. A. Before percutaneous coronary intervention, total occlusion at proximal left anterior descending artery, thrombus grade 5, and TIMI flow 1. B. After percutaneous coronary intervention, TIMI flow 1 with residual thrombus in LAD and shifting thrombus to distal left circumflex artery. C. After 4 months follow-up, normal coronary arteries without any apparent atherosclerotic lesion.

The patient was diagnosed with extensive anterior STEMI (Killip I, TIMI 3/14), nephrotic syndrome, and community-acquired pneumonia. Coronary angiography (CAG) revealed total occlusion of proximal left anterior descending (LAD) artery, thrombus grade 5, and TIMI flow 1 ( Figure 2A). Plain old balloon angioplasty (POBA) was planned for the LAD. However, despite multiple attempts of extensive POBA, CAG showed TIMI flow 1 with residual thrombus in the LAD and a shifting thrombus to the distal left circumflex artery (LCx) ( Figure 2B). Given these findings, it was decided to defer further interventions and proceed with medical treatment using intravenous antiplatelet infusion and anticoagulant therapy.

The patient received eptifibatide infusion, heparinization, oral dual antiplatelet therapy (aspirin and ticagrelor), an ACE inhibitor, statin, nitrate, and antibiotic. The following day, he reported no chest pain and ECG did not show any dynamic ST-T changes ( Figure 1B). Steroid therapy was initiated and he was discharged in stable condition. Four months later, a follow-up **CAG** was performed in the absence of any symptoms. The result showed normal coronary arteries without any apparent atherosclerotic lesions ( Figure 2C).

### Case 2

A 30-year-old Sundanese male presented with a chronic leg wound that had persisted for six months, accompanied by resting pain. He initially experienced claudication, reporting pain in both legs while walking long distances over the past six years. The patient had been diagnosed with NS 12 years ago, but didn’t take medication regularly. He was a heavy smoker, smoking one pack of cigarettes per day. He denied history of hypertension or diabetes mellitus.

**Figure 3.**
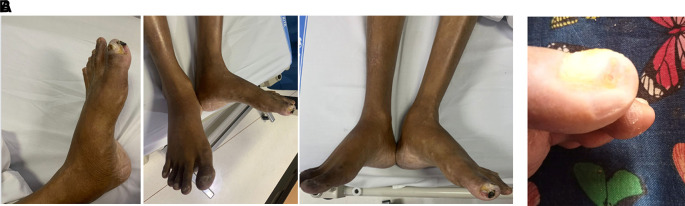
Clinical image of 2 ^nd^ patient. A. Gangrene was seen on the left toe at presentation. B. Resolution of gangrene after 3 weeks follow-up.

**Figure 4.**
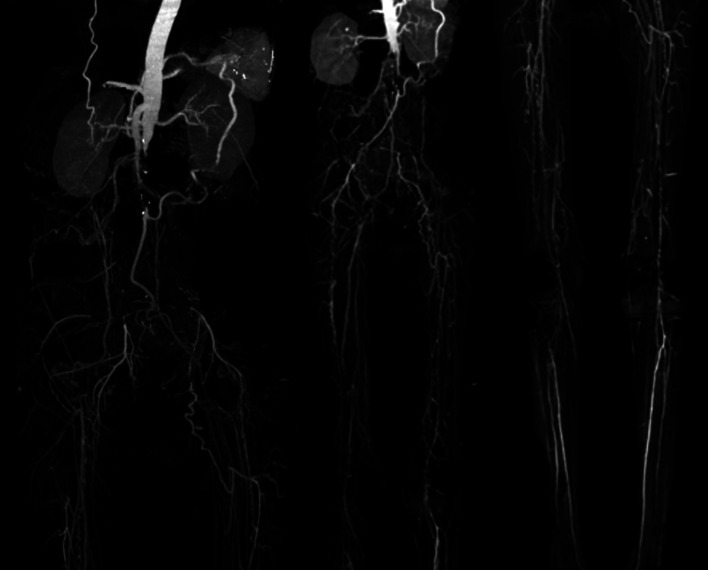
Lower extremity CT Angiography of 2 ^nd^ patient. Occlusion with thrombus in abdominal aorta starting from 2 cm below renal artery until bilateral superficial femoral artery, distal flow filled from collateral from branch of coeliac trunk and branch of superior mesenteric artery.

On physical examination, his vital signs were within normal limits. Hist extremities were cold, non-palpable dorsalis pedis artery pulses bilaterally, and he had gangrene on his left toe ( Figure 3A). Significant laboratory findings were erythrocyte sedimentation rate of 99 mm/hour, D-dimer of 3250 ng/mL, fibrinogen of 734 mg/dL, albumin of 0.8 g/dL, total cholesterol of 347 g/dL, LDL of 257 g/dL, HDL of 54 g/dL, triglyceride of 278 g/dL, +3 urinary protein with 24-hour urinary protein of 19840 mg/24 hour. The ankle brachial index (ABI) was 0.25 on the left and 0.33 on the right. Lower extremity duplex ultrasound (DUS) findings were consistent with CT Angiography (CTA), which revealed a thrombotic occlusion of the abdominal aorta, beginning 2 cm below the renal artery and extending to the bilateral superficial femoral artery (SFA). Distal perfusion was maintained through collateral branches of celiac trunk and superior mesenteric artery ( Figure 4).

The patient was diagnosed with CLTI (Rutherford III-5, WIFi Score 2-3-0) due to aortio-iliaca occlusive disease (TASC II type D lesion) and nephrotic syndrome. Unfortunately, he declined any interventional therapy. As an alternative, he was managed with albumin transfusion, methylprednisolone therapy using titration method, heparinization, clopidogrel, lumbrokinase, simvastatin, diltiazem, candesartan, and other supportive symptomatic medication. After five days of treatment, the patient was discharged with reduced leg pain. His albumin level increased to 2.9 g/dL, and his 24-hour urinary protein decreased to 5685 mg/24 hour. His prescribed discharge medications included methylprednisolone 16 mg three times daily, clopidogrel, simvastatin, candesartan, and diltiazem. At the three-week follow-up, he reported significant relief from leg pain, and the gangrene had resolved ( Figure 3B).

### Case 3

A 32-year-old Javanese male presented with chief complaints of leg pain that had persisted for one year. Initially the pain occurred only while walking for distances, but it progressively worsened, leading to resting pain over the past month. The patient had a nine-year history of NS confirmed by kidney biopsy showing focal segmental glomerulosclerosis. He denied history of hypertension, diabetes, or smoking. At the time of presentation, he took 2 × 360 mg mycophenolic acid and 1 × 8 mg methylprednisolone daily.

**Figure 5.**
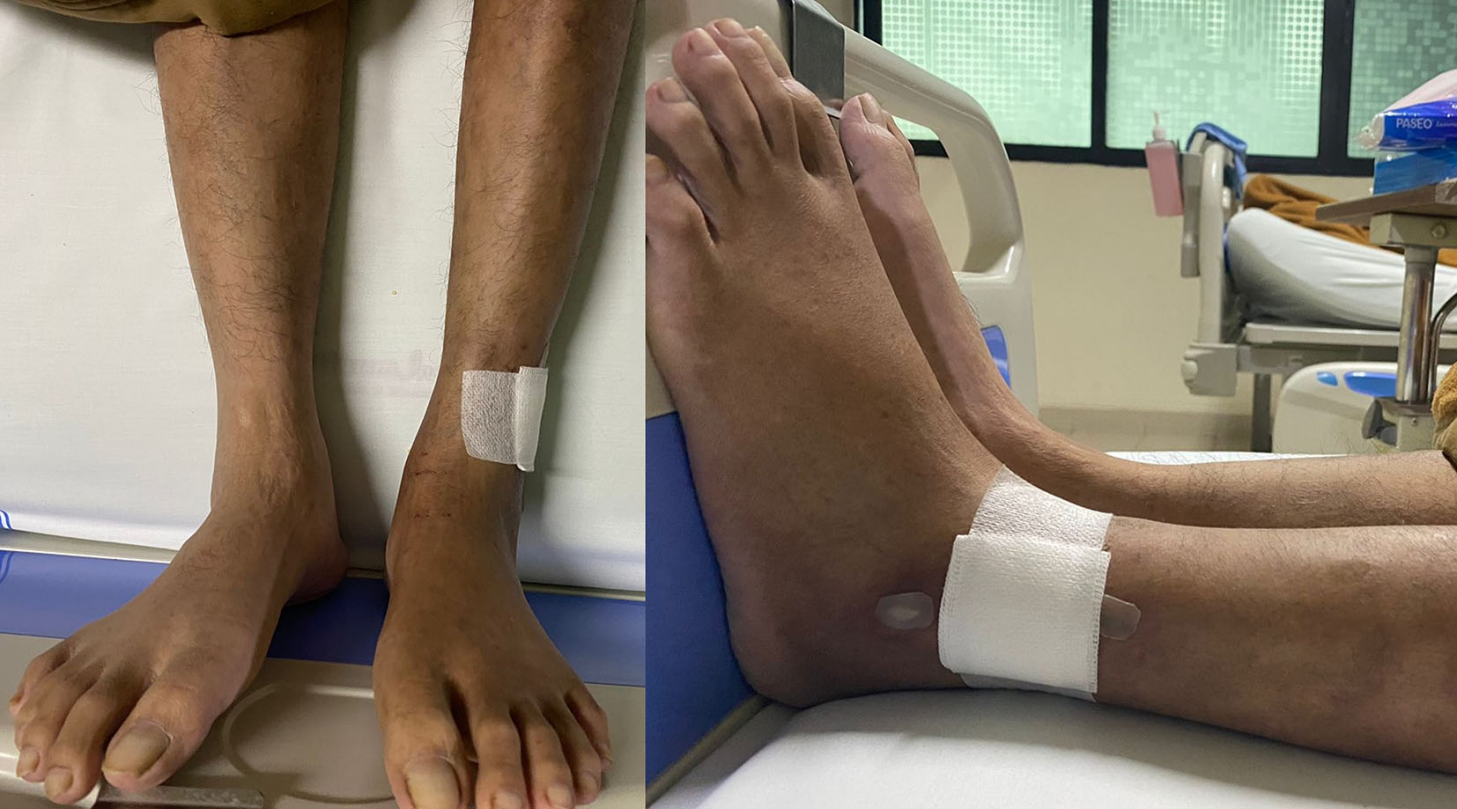
Clinical image of 3 ^rd^ patient. Ulcer (covered by bandage), hair loss, and atrophy were seen on the left leg.

**Figure 6.**
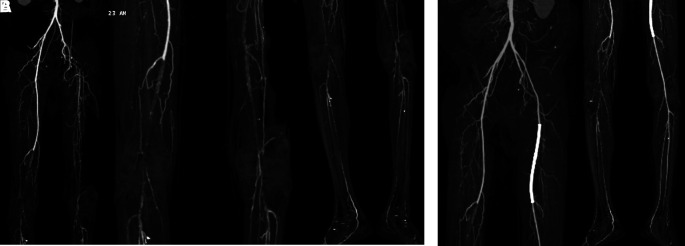
Lower extremity CT Scan Angiography of 3 ^rd^ patient. A. Pre-intervention, occlusion at level of left external iliac artery and 1/3 distal of right superficial femoral artery with prominent plaque calcification. B. Before 2 ^nd^ intervention, positive flow until distal of the left leg with patent stent.

His vital signs were within normal limits. Physical examination revealed an ulcer, hair loss, and muscle atrophy on the left leg ( Figure 5). Significant laboratory findings were D-Dimer of 2990 ng/mL, total cholesterol of 233 g/dL, LDL of 187 g/dL, triglycerides of 164 g/dL, and urine albumin of 413 mg/L. His serum albumin was normal (184 g/dL). His right ABI was 0.5 on left was 0.33. Lower extremity DUS and CTA showed occlusion at level of left external iliac artery and distal one-third of the right SFA with prominent plaque calcification ( Figure 6A).

**Figure 7.**
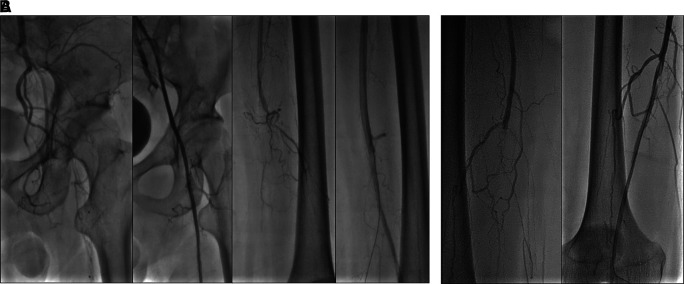
Percutaneous transluminal angioplasty procedure of 3 ^rd^ patient. A. First intervention, contrast flow until distal of left leg artery after percutaneous transluminal angioplasty. B. Second intervention, contrast flow until distal of right leg artery after percutaneous transluminal angioplasty.

The patient was diagnosed with CLTI with an ulcer on the left leg (Rutherford III-5, WIFi Score 1-3-0), chronic limb ischemia on the right leg (Rutherford I-3, WIFi Score 0-2-0), TASC II type D lesion, and nephrotic syndrome. The patient underwent heparinization and two episodes of percutaneous transluminal angioplasty (PTA). First procedure with POBA performed on the left Iliac Artery and SFA, followed by placement of a 6.0 × 120 mm drug-eluting stent (DES) overlapping with a 6.0 × 80 mm DES (Boston Scientific, Marlborough, MA, USA) at the SFA ( Figure 7A). Second procedure was POBA performed on the mid-to-distal right SFA ( Figure 7B). CTA after the first procedure ( Figure 6B) and angiography after second procedure with lower extremity DUS confirmed positive blood flow until the distal vessel of both lower limbs. The patient was discharged without any complaints and was prescribed rivaroxaban, clopidogrel, aspirin, simvastatin, mycophenolic acid, and methylprednisolone as part of his routine medication. He was also educated on exercise therapy to improve circulation and prevent disease progression.

## Discussion

NS related ATE cases are rare and primarily reported in case reports or small case series. ^10^ According to a publication by Mahmoodi, et al. ^11^ which includes 298 consecutive NS patients, the annual incidence of ATE was 1.48%. The most common first ATE presentation in NS patients was myocardial infarction (MI) (44%), followed by unstable angina pectoris (14%), peripheral artery disease (14%), ischemic stroke (11.5%), cerebral transient ischemic attack (11.5%), amaurosis fugax (2%), and aorta thrombosis (2%). Another cohort study, which includes 3967 adults with first-time NS reported a 1-year ATE risk of 4.2% (95% confidence interval [CI] 3.6-4.8) and 10-year risk of 14.0% (95% CI 12.8-15.2), with the risk for MI reaching as high as 6%. ^12^ Additionaly, a single center case series found that among 1,800 MI admissions, eight patients had NS-associated MI. ^13^ Furthermore, a cross-sectional study comparing the prevalence of PAD in in 100 children with NS and 100 healthy controls found significantly higher rates in NS patients (44.0% vs. 6.0%, p < 0.001). ^14^

The pathophysiology of ATE in NS remains incompletely understood. It has been postulated that plasma protein alteration contribute to coagulation and fibrinolysis disturbance, increased aggregation of platelet, low albumin plasma, hyperviscosity, and dyslipidemia. ^11^^,^^15^ Chronic excessive proteinuria combined with long-term abnormalities in hemostasis and lipid profiles, play a significant role in this process. ^16^ There are three proposed mechanisms related to the hypercoagulable state in NS patients. First, enhanced coagulation related to low molecular weight protein loss such as factors IX, XI, and XII from urine, thereby the liver increased synthesis of factors II, VII, VIII, X, XIII, and fibrinogen to compensate the hypoalbuminemia state. Second, decreased anticoagulation such as Antithrombin III that has been observed in low serum albumin condition. Third, fibrinolytic system imbalance related to decreased levels of plasminogen and raised levels of plasminogen activator that correlate with the degree of hypoalbuminaemia. Additionally, systemic inflammation marked by upregulation of circulating proinflammatory cytokines such as interleukin-1β, tumor necrosis factor-α, and phospholamban may further contribute to the pathophysiology. ^17^ These mechanisms collectively lead to endothelial dysfunction as “risk of the risk factors” due to endothelial injury which associated with an increased likelihood of future cardiovascular events in NS patients. ^18^

Our first and second patient were in the acute phase of NS with significantly low serum albumin levels, predisposing them to thromboembolic events. Literature suggests thromboses are more common when plasma albumin levels drop below 2 g/dL. ^19^ However, our third patient had good control of the disease, known from relatively normal serum albumin. We suspected that long-term corticosteroid use by our patient promotes a hypercoagulable state since it increased factors II, V, VII, IX, X, and XII and fibrinogen, thereby increasing risk for thrombosis. ^20^ Hyperlipidemia in all of our patients is also known to be a risk factor for thrombosis since it induced platelet hyperaggregability. ^21^ A key question is whether these cases represent true atherosclerotic events or purely thromboembolic phenomena. The first patient, diagnosed with STEMI, underwent CAG, which revealed a high thrombus burden (HTB) without significant underlying atherosclerotic plaques. This suggests a primary thromboembolic event rather than classic atherosclerosis. A study by Xie et al. supports this finding, showing that NS patients presenting with ACS more commonly exhibit acute coronary thrombosis rather than atheromatous plaques. ^16^ In second case, the presence of occlusive thrombi without extensive calcification also suggests a predominant thrombotic mechanism rather than chronic atherosclerosis. However, the third case involved occlusion with prominent plaque calcification indicating potential role of atherosclerosis. Further studies should investigate this phenomenon and explore the potential relationship between albumin levels with the patophysiology of ATE in NS.

Currently, there is no consensus according to the management of thromboembolic complications related to NS. ^22^ The management mainly follows the available guidelines and depends on the location and severity of thrombotic events. In our first patient, the likely mechanism of myocardial infarction was coronary thrombosis, as demonstrated by HTB in CAG. A thrombus in coronary artery with a score of ≥4 is defined as a HTB, which deferred stent placement has been associated with a better outcome. ^23^ Pharmacological therapies that are used for HTB treatment include antiplatelet, anticoagulant, thrombolytic, statins, and vasodilators.

In CLTI, existing evidence strongly support for selective revascularization based on specific clinical and anatomical criteria. ^5^ Endovascular interventions in CLTI rely upon the ability to cross the Femoro-Popliteal lesion, utilizing techniques for vessel preparation and definitive therapy. ^24^ Unfortunately, there are limited publications providing guidance on selecting specific endovascular strategies for CLTI patients. A recent study demonstrated that surgical-first strategy is associated with 32% lower risk of major adverse limb events or death compared to an endovascular first approach in the setting of patients with good-quality great saphenous vein conduit. ^25^ However, in those without suitable great saphenous vein, the overall eficacy and safety of both approaches appear similar. Tho, the decision to choose revascularization technique should be individualized and involve a multidisciplinary team appraoch. CLTI patients are recommended to receive pharmacological therapy with antiplatelet and moderate-to-high-intensity statin therapy to reduce the risk of major adverse cardiovascular events. ^24^ For patients that are not suitable for revascularization, there are few options for non revascularization interventions, pharmacotherapy, and conservative management. We would like to choose an endovascular approach for our second and third patient, however, the second patient refused any intervention, so we optimized the pharmacological therapy. Both of our patients achieved significant improvement in the disease.

Treatment of NS patients with immunosuppressive therapy combined with steroids can reduce disease activity, which reduced approximately 40% risk of progression to end-stage renal disease compared to no treatment or supportive treatment alone. ^26^ Prophylaxis for thromboembolism also can be given to NS patients depending on histological subtype, bleeding risk, and serum albumin level, which are received by our patients. ^27^

Our study has a few limitations, including a small sample size and a lack of long-term follow-up data. We recommend future structured studies to analyze ATE in NS patients with a larger sample size, a control group for comparison, and a longer follow-up period to determine whether NS significantly contributes to the disease.

## Conclusion

NS is a significant risk factor for ATE driven by a hypercoagulable state, endothelial dysfunction, and atherosclerosis. Given the high thrombotic risk, NS patients should undergo routine cardiovascular screening, particularly those with severe hypoalbuminemia and hyperlipidemia. Preventive strategies, including thromboprophylaxis in high-risk individuals, aggressive lipid management, and careful monitoring of corticosteroid therapy are essential. Treatment should be tailored to the underlying mechanism, with deferred stenting considered in high thrombus burden STEMI cases and a multidisciplinary approach for CLTI, integrating pharmacologic therapy and revascularization when appropriate. Long-term follow-up is critical to prevent recurrent thromboembolic events, and further research is needed to optimize individualized risk assessment and treatment strategies in this high-risk population.

## Consent

Written informed consent for publication of their clinical details and clinical images was obtained from the patients.
