# Supplementary material for: Coexisting nephrotic syndromes influences in st elevation myocardial infarction patient and chronic limb-threatening ischemia patient: is there any correlation?
Source: F1000Res. 2025 Mar 31;12:538. Originally published 2023 May 23. [Version 2] doi: 10.12688/f1000research.134021.2 (PMC11973628; doi:10.12688/f1000research.134021.2)
Supplement: Peer review history

# Reviewer report: Reviewer response for version 2

**DOI:** 10.5256/f1000research.179594.r374569

**Published:** 2025-04-02

**Authors:** Seung-Woon Rha, Markz Sinurat

**Affiliations:** 1 Cardiovascular Center, Korea University Guro Hospital, Seoul, South Korea; 2 Cardiovascular Department, Universitas Padjadjaran (Ringgold ID: 61809), Bandung, West Java, Indonesia

No further comments, the authors had provided great insight into the revised document.

Are enough details provided of any physical examination and diagnostic tests, treatment given and outcomes?

Partly

Is the background of the cases’ history and progression described in sufficient detail?

Partly

Is sufficient discussion included of the importance of the findings and their relevance to future understanding of disease processes, diagnosis or treatment?

Partly

Is the conclusion balanced and justified on the basis of the findings?

Partly

Reviewer Expertise:

Cardiovascular

We confirm that we have read this submission and believe that we have an appropriate level of expertise to confirm that it is of an acceptable scientific standard.

# Reviewer report: Reviewer response for version 1

**DOI:** 10.5256/f1000research.147046.r368203

**Published:** 2025-03-11

**Authors:** Seung-Woon Rha, Markz Sinurat

**Affiliations:** 1 Cardiovascular Center, Korea University Guro Hospital, Seoul, South Korea; 2 Cardiovascular Department, Universitas Padjadjaran (Ringgold ID: 61809), Bandung, West Java, Indonesia

Suggestion: Major revision

 General comment)

 First, I would like to remark on the strengths of your study.

 Relevance and Novelty

- The paper addresses a unique and understudied relationship between nephrotic syndrome (NS), ST-elevation myocardial infarction (STEMI), and chronic limb-threatening ischemia (CLTI).
- It provides real-world clinical insights into cardiovascular complications in young adults with NS, which is valuable for clinical practice.

Case-Based Approach

- The case series format helps illustrate the clinical presentation, diagnostic process, and treatment strategies.
- The inclusion of angiography and clinical imaging data strengthens the discussion and helps visualize disease progression.

Pathophysiological Insight

- The discussion of thromboembolic mechanisms in NS patients is detailed, citing hypercoagulability, dyslipidemia, and corticosteroid-induced thrombosis.
- It appropriately references prior studies that link NS to arterial thromboembolism.

However, several aspects of this study need to be clarified.

 Major)

 1. Language and Clarity Issues

- The writing has grammatical errors and awkward phrasing, which affects readability.
  Example:
  - "NS is a risk factor for STEMI and CLTI due to thrombosis and/or atherosclerotic processes." → Needs refinement.
  - Many sentences are long and difficult to follow, which can be clarified.
  - Figure 2. Coroangiography?--> Coronary Angiography

2. Study Design Limitations

- Lack of Comparative Data
  - The paper does not compare the NS patients with a control group (i.e., STEMI or CLTI patients without NS). This limits the ability to conclude whether NS significantly contributes to arterial thromboembolism.
- Limited Case Number
  - Three cases are too few to establish a strong correlation between NS and STEMI/CLTI. A larger retrospective analysis or case-control study would be more convincing.
- Lack of Statistical Analysis
  - The discussion is mostly descriptive, without any attempt to quantify risks or assess statistical significance. Even a basic comparison (e.g., incidence rate of STEMI/CLTI in NS patients vs. general population) would add strength.

3. Discussion Gaps

- Unclear Mechanism of Atherothrombosis in NS
  - While hypercoagulability is discussed, the role of endothelial dysfunction and chronic inflammation in NS-related atherosclerosis is underexplored.
  - Are the cases true atherosclerotic events or purely thromboembolic? This should be clarified.
- Alternative Therapies for CLTI
  - The reviewer suggests discussing surgical options for CLTI (e.g., bypass surgery). Currently, only endovascular and pharmacological management are considered.
- Follow-up Data
  - While the first patient had a follow-up coronary angiography, the long-term outcomes of other patients are missing.
  - Did CLTI patients maintain symptom improvement? Any reintervention?

4. Weak Conclusion

- The conclusion does not add much beyond the discussion and lacks a strong clinical takeaway.
  - Instead of just stating that NS increases the risk of STEMI and CLTI, the authors should provide clear recommendations for screening, prevention, and treatment strategies.

Are enough details provided of any physical examination and diagnostic tests, treatment given and outcomes?

Partly

Is the background of the cases’ history and progression described in sufficient detail?

Partly

Is sufficient discussion included of the importance of the findings and their relevance to future understanding of disease processes, diagnosis or treatment?

Partly

Is the conclusion balanced and justified on the basis of the findings?

Partly

Reviewer Expertise:

Cardiovascular

We confirm that we have read this submission and believe that we have an appropriate level of expertise to state that we do not consider it to be of an acceptable scientific standard, for reasons outlined above.

# Response

**Published:** 2025-03-20

**Authors:** Taofan Taofan

**Affiliations:** National Cardiovascular Center Harapan Kita, Indonesia

Dear Reviewers,

 We sincerely appreciate your time and effort in reviewing our manuscript. Your valuable feedback has helped us improve the clarity, depth, and overall quality of our work. Below, we have addressed each of your comments in detail and have made the necessary revisions accordingly. We hope these modifications enhance the manuscript and align with your expectations.

**1. Language and Clarity Issues**

- **The writing has grammatical errors and awkward phrasing, which affects readability.**
** Example:**
  - **"NS is a risk factor for STEMI and CLTI due to thrombosis and/or atherosclerotic processes." → Needs refinement.**
  - **Many sentences are long and difficult to follow, which can be clarified.**
  - **Figure 2. Coroangiography?--> Coronary Angiography**

**Author Response:** We’ve made substantial revision to the grammar and phrasing.

**2. Study Design Limitations**

- **Lack of Comparative Data**
  - **The paper does not compare the NS patients with a control group (i.e., STEMI or CLTI patients without NS). This limits the ability to conclude whether NS significantly contributes to arterial thromboembolism.**
- **Limited Case Number**
  - **Three cases are too few to establish a strong correlation between NS and STEMI/CLTI. A larger retrospective analysis or case-control study would be more convincing.**

**Author Response: **Due to the limited case numbers, we’re unable to conduct a study with other designs. Reviewer’s concern about this part has been added to the limitation of our study.

- **Lack of Statistical Analysis**
  - **The discussion is mostly descriptive, without any attempt to quantify risks or assess statistical significance. Even a basic comparison (e.g., incidence rate of STEMI/CLTI in NS patients vs. general population) would add strength.**

**Author Response: **Following reviewer’s suggestion, we’ve added more of these into the discussion.

 “NS related ATE cases are rare and primarily reported in case reports or small case series. ^10^ According to a publication by Mahmoodi, et al. ^11^ which includes 298 consecutive NS patients, the annual incidence of ATE was 1.48%. The most common first ATE presentation in NS patients was myocardial infarction (MI) (44%), followed by unstable angina pectoris (14%), peripheral artery disease (14%), ischemic stroke (11.5%), cerebral transient ischemic attack (11.5%), amaurosis fugax (2%), and aorta thrombosis (2%). Another cohort study, which includes 3967 adults with first-time NS reported a 1-year ATE risk of 4.2% (95% confidence interval [CI] 3.6-4.8) and 10-year risk of 14.0% (95% CI 12.8-15.2), with the risk for MI reaching as high as 6%. ^12 ^Additionaly, a single center case series found that among 1,800 MI admissions, eight patients had NS-associated MI.. ^13^  Furthermore, a cross-sectional study comparing the prevalence of PAD in in 100 children with NS and 100 healthy controls found significantly higher rates in NS patients (44.0% vs. 6.0%, p < 0.001). ^14^”

**3. Discussion Gaps**

- **Unclear Mechanism of Atherothrombosis in NS**
  - **While hypercoagulability is discussed, the role of endothelial dysfunction and chronic inflammation in NS-related atherosclerosis is underexplored.**

**Author Response: **Further discussion regarding the pathophysiology behind ATE related NS has been added and includes inflammation and endhotelial dysfunction.

 “Additionally, systemic inflammation marked by upregulation of circulating proinflammatory cytokines such as interleukin-1β, tumor necrosis factor-α, and phospholamban may further contribute to the pathophysiology. ^17^ These mechanisms collectively lead to endothelial dysfunction as “risk of the risk factors” due to endothelial injury which associated with an increased likelihood of future cardiovascular events in NS patients. ^18”^

  

-
  - **Are the cases true atherosclerotic events or purely thromboembolic? This should be clarified.**

**Author Response: **We’ve clarified this concern in the discussion.

 “A key question is whether these cases represent true atherosclerotic events or purely thromboembolic phenomena. The first patient, diagnosed with STEMI, underwent CAG, which revealed a high thrombus burden (HTB) without significant underlying atherosclerotic plaques. This suggests a primary thromboembolic event rather than classic atherosclerosis. A study by Xie et al. supports this finding, showing that NS patients presenting with ACS more commonly exhibit acute coronary thrombosis rather than atheromatous plaques. ^16^ In second case, the presence of occlusive thrombi without extensive calcification also suggests a predominant thrombotic mechanism rather than chronic atherosclerosis. However, the third case involved occlusion with prominent plaque calcification indicating potential role of atherosclerosis. Further studies should investigate this phenomenon and explore the potential relationship between albumin levels with the patophysiology of ATE in NS.”

  

- **Alternative Therapies for CLTI**
  - **The reviewer suggests discussing surgical options for CLTI (e.g., bypass surgery). Currently, only endovascular and pharmacological management are considered.**

**Author Response: **Alternative therapy for CLTI has been added including surgical-first strategy.

 “A recent study demonstrated that surgical-first strategy is associated with 32% lower risk of major adverse limb events or death compared to an endovascular first approach in the setting of patients with good-quality great saphenous vein conduit. ^25^ However, in those without suitable great saphenous vein, the overall eficacy and safety of both approaches appear similar. Tho, the decision to choose revascularization technique should be individualized and involve a multidisciplinary team appraoch.”

  

- **Follow-up Data**
  - **While the first patient had a follow-up coronary angiography, the long-term outcomes of other patients are missing.**
  - **Did CLTI patients maintain symptom improvement? Any reintervention?**

**Author Response: **Unfortunately, we can’t provide long-term follow up data due to our center is a national referral hospital and those patients in our case were from another city. They do the follow-up in their own hometown. This concern also has been addressed in the limitations of our study.

**4. Weak Conclusion**

- **The conclusion does not add much beyond the discussion and lacks a strong clinical takeaway.**
  - **Instead of just stating that NS increases the risk of STEMI and CLTI, the authors should provide clear recommendations for screening, prevention, and treatment strategies.**

**Author Response: **Stronger conclusions have been provided

 “NS is a significant risk factor for ATE driven by a hypercoagulable state, endothelial dysfunction, and atherosclerosis. Given the high thrombotic risk, NS patients should undergo routine cardiovascular screening, particularly those with severe hypoalbuminemia and hyperlipidemia. Preventive strategies, including thromboprophylaxis in high-risk individuals, aggressive lipid management, and careful monitoring of corticosteroid therapy are essential. Treatment should be tailored to the underlying mechanism, with deferred stenting considered in high thrombus burden STEMI cases and a multidisciplinary approach for CLTI, integrating pharmacologic therapy and revascularization when appropriate. Long-term follow-up is critical to prevent recurrent thromboembolic events, and further research is needed to optimize individualized risk assessment and treatment strategies in this high-risk population.”

 Once again, we are grateful for your insightful feedback, which has helped refine our manuscript. We believe the revisions have strengthened the clarity and clinical relevance of our work. We look forward to your further guidance and hope that the revised version meets your expectations. Thank you for your time and consideration.

# Reviewer report: Reviewer response for version 1

**DOI:** 10.5256/f1000research.147046.r225876

**Published:** 2023-12-18

**Authors:** Yopie Afriandi Habibie

**Affiliations:** 1 Universitas Syiah Kuala, Banda Aceh, Indonesia

Congratulations to all the authors for writing a well-written article. In the article, the author shares their experience with three Nephrotic Syndrome patients. However, it would be helpful to add some suggestions about alternative therapies for CLTI patients in the Discussion Chapter, including surgical measures that can be performed for cases unsuitable for endovascular treatment.

Are enough details provided of any physical examination and diagnostic tests, treatment given and outcomes?

Yes

Is the background of the cases’ history and progression described in sufficient detail?

Yes

Is sufficient discussion included of the importance of the findings and their relevance to future understanding of disease processes, diagnosis or treatment?

Yes

Is the conclusion balanced and justified on the basis of the findings?

Yes

Reviewer Expertise:

Cardiovascular Medicine, Vascular Surgery, Surgery, Endovascular Theraphy, Thoracic Surgery, Cardiac Surgery

I confirm that I have read this submission and believe that I have an appropriate level of expertise to confirm that it is of an acceptable scientific standard.

## References

[1] : Outcomes of a bypass-first strategy in chronic limb-threatening ischemia based on the Global Vascular Guidelines. *J Vasc Surg* .2023;77(1) : 10.1016/j.jvs.2022.06.103 201-207 10.1016/j.jvs.2022.06.103 35934216
